# Micromolding of Amphotericin-B-Loaded Methoxyethylene–Maleic Anhydride Copolymer Microneedles

**DOI:** 10.3390/pharmaceutics14081551

**Published:** 2022-07-26

**Authors:** Sina Azizi Machekposhti, Alexander K. Nguyen, Lyndsi Vanderwal, Shane Stafslien, Roger J. Narayan

**Affiliations:** 1 Joint UNC/NCSU Department of Biomedical Engineering, North Carolina State University, Raleigh, NC 27695, USA; sazizim@ncsu.edu (S.A.M.); aknguye2@ncsu.edu (A.K.N.); 2 Coatings and Polymeric Materials, North Dakota State University, Fargo, ND 58102, USA; lyndsijo@live.com (L.V.); shane.stafslien@ndsu.edu (S.S.)

**Keywords:** microneedles, amphotericin B, transdermal drug delivery, fungus

## Abstract

Biocompatible and biodegradable materials have been used for fabricating polymeric microneedles to deliver therapeutic drug molecules through the skin. Microneedles have advantages over other drug delivery methods, such as low manufacturing cost, controlled drug release, and the reduction or absence of pain. The study examined the delivery of amphotericin B, an antifungal agent, using microneedles that were fabricated using a micromolding technique. The microneedle matrix was made from Gantrez^TM^ AN-119 BF, a benzene-free methyl vinyl ether/maleic anhydride copolymer. The Gantrez^TM^ AN-119 BF was mixed with water; after water evaporation, the polymer exhibited sufficient strength for microneedle fabrication. Molds cured at room temperature remained sharp and straight. SEM images showed straight and sharp needle tips; a confocal microscope was used to determine the height and tip diameter for the microneedles. Nanoindentation was used to obtain the hardness and Young’s modulus values of the polymer. Load–displacement testing was used to assess the failure force of the needles under compressive loading. These two mechanical tests confirmed the mechanical properties of the needles. In vitro studies validated the presence of amphotericin B in the needles and the antifungal properties of the needles. Amphotericin B Gantrez^TM^ microneedles fabricated in this study showed appropriate characteristics for clinical translation in terms of mechanical properties, sharpness, and antifungal properties.

## 1. Introduction

The drug delivery administration route is an important topic for optimizing drug efficacy. Each administration route is associated with various benefits and shortcomings; the most suitable route must be selected for each drug type. The oral route is convenient and pain-free [1]; due to first-pass metabolism and poor absorption, the desired serum level may not be obtained [2]. Injections are painful, require trained staff, and produce sharp biohazard waste [3]. Novel delivery methods may improve efficacy and adherence to drug therapies [4]. Local delivery approaches have been used to deliver therapeutic agents and vaccines to the skin, which is the largest organ [5].

The stratum corneum is the outermost layer of human skin; it exhibits a thickness of 10–15 µm [6] and serves as a barrier to the transepidermal delivery of many types of drugs. Microneedles can be painless if they do not interact with the unmyelinated nerve endings that detect pain or the mechanoreceptors that detect pressure in the dermis [7,8].

Microneedles made from biodegradable polymers are nearly painless and require no trained staff for local release of therapeutic agents; these devices leave no sharp medical waste after use [1,9,10,11].

Various biocompatible polymers, such as polylactic acid (PLA) [12], polyvinylpyrrolidone (PVP) [13], poly(lactic-co-glycolic acid) (PLGA) [14], poly(glycolic acid) (PGA) [15], and carboxymethyl cellulose (CMC) [16], have been used for making microneedles. Gantrez^TM^ AN-119 BF is a synthetic biodegradable copolymer that contains methyl vinyl ether and maleic anhydride units [17]. When Gantrez^TM^ AN-119 BF is dissolved in water, the anhydride side chain hydrolyzes and produces free diacid solutions [18,19]. The mechanical strength of the polymer can be increased through the incorporation of cross-linking agents [20]. Microneedles must possess appropriate mechanical properties in order to pierce the outermost layer of the skin, the stratum corneum. After penetrating the stratum corneum, the biocompatible polymer is dissolved, and the drug is released. Various medical conditions, including influenza, acne, and diabetes, are able to be treated using microneedle therapies [21]. Polymers used in microneedles must exhibit certain manufacturing characteristics. The monomer must flow into the tiny bores of the molds; moreover, attachment of the polymer to the walls needs to be prevented by anti-stick materials or alterations to the mold. The reaction the monomer or polymer might have with the drug must also be considered.

In this study, the microneedle master structure was created by two-photon polymerization (2PP); the master structure was used for making molds from polydimethylsiloxane (PDMS). Molding was performed at different temperature situations to obtain straight microneedles. Amphotericin B was added to the dissolved Gantrez^TM^ in water; this solution was stirred for water evaporation and subjected to micromolding. The replicas of the master structure were made from Gantrez^TM^ AN-119 BF/amphotericin B with two different weight ratios; mechanical testing was used to select the optimum ratio. Nanoindentation and compression testing were used to assess the mechanical properties of the microneedles. Confocal microscopy was used for imaging of the needles. Raman spectroscopy, X-ray photoelectron spectroscopy (XPS), high-performance liquid chromatography (HPLC), and FTIR were used to assess the chemical properties of the microneedles. Finally, an in vitro study was used to examine the antifungal properties of the microneedles.

## 2. Materials and Methods

### 2.1. Master Structure Fabrication

The master structure from a previous study [22] was used to fabricated microneedles in this study. In brief, 1 cm diameter hexagonal array master structures were fabricated using the two-photon polymerization (2PP) approach. The base diameter of conical microneedles was 250 μm; the microneedles exhibited nominal heights of 500, 750, or 1000 μm. The nominal spacing of the needles was 295.45, 361.11, or 423.91 μm for the microneedles with nominal heights of 500, 750, or 1000 μm, respectively. A Mai-Tai titanium:sapphire femtosecond laser (Newport Corporation, Irvine, CA, USA) was used to generate laser pulses (780 nm wavelength, <80 fs pulse duration, 80 MHz repetition rate, M2 < 1.1). The laser beam passed through (a) a half-waveplate/polarizing beamsplitter pair for manual power control, (b) an acousto-optical modulator (Gooch & Housego, Ilminster, UK) for computer power control, (c) a pinhole spatial filter for removing high-order spatial modes and to expand the beam to 1 cm diameter, (d) a galvanoscanner for fine laser positioning, and (e) a 20×, 0.5 NA microscope objective. A laser speed of 50 mm/s, hatching of 1.5 μm, and layer height of 3 μm were used to process the master structures [22].

### 2.2. Microneedle Fabrication

Microneedles were fabricated using a micromolding technique. Two types of PDMS molds were fabricated in this study. In the first type, PDMS (Sylgard 184, Dow Corning, Midland, MI, USA) was placed in a 60 °C furnace for five hours for polymerization; in the second type, PDMS (Sylgard 184, Dow Corning, Midland, MI, USA) was kept at room temperature for two days. After the molds were fabricated, Gantrez^TM^ (Gantrez^TM^ AN-119 BF, Ashland Global Holdings Inc., Wilmington, DE, USA) was dissolved in deionized water (80% *w*/*w*), agitated for two days, degassed under vacuum, and sonicated to facilitate entry of material into the bores of the mold. Amphotericin B powder was added to the matrix for the last 12 h. Simultaneous vacuuming and sonication were helpful for reducing the vacuum pressure and producing sharper needles. When using the mold with material at room temperature, water was evaporated, and the matrix was polymerized. Microneedles with two drug ratios (4% and 8%) were fabricated in this study. Since amphotericin B is insoluble in water, it is in the form of a suspension solution with Gantrez^TM^ powder.

### 2.3. Variable Pressure Scanning Electron Microscopy

An S-3200 variable pressure scanning electron microscope (Hitachi, Tokyo, Japan) was used to obtain images from the microneedles. Microneedles were sputter-coated with a 60% gold-40% palladium layer using a Technics Hummer II system (Anatech, Battle Creek, MI, USA) for two minutes [21].

### 2.4. Mechanical tests

#### 2.4.1. Nanoindentation

Nanoindentation was used to obtain hardness and Young’s modulus values from the microneedle material. A Ubi-1 Nanoindenter (Hysitron, Minneapolis, MN, USA) was used for this study; a Berkovich-type tip was used for analysis of the materials. The maximum force was 1000 μN; for each test, a loading time of 20 s, a dwell time of 10 s at maximum load, and an unloading time of 20 s were used. Through Oliver–Pharr analysis of the unloading curves, the hardness and Young’s modulus values were calculated [23].

#### 2.4.2. Compressive Loading of Microneedles

An ElectroForce^®^ 3100 instrument (Bose Corporation, Framingham, MA, USA) was used to assess the fracture properties of the microneedles when loaded under compression. Microneedles were placed on the bottom platen of the instrument using double sided tape. The top platen was actuated in three different steps. The first step was loading at 0.005 N/s to −0.015 N. This step was used to ensure that the microneedles were in contact with the probe. The second step involved a dwell time of 10 s. The last step involved displacement at 0.0025 mm/second to −1.5 mm. The negative value for displacement and load were used to indicate the compressive nature of the loading process [24]. These tests were repeated for 5 microneedles of each type.

### 2.5. 3D Laser Scanning Confocal Microscopy

A VK-X250 3D laser scanning confocal microscope (Keyence, Tokyo, Japan) was used for imaging of the microneedle dimensions and surface topography. In this study, the laser was rastered in the XY pattern; the steps in the Z-direction were 0.5 nm. This microscope was equipped with laser confocal optics to measure the field depth. Keyence imaging was used to assess the sharpness, height, and base diameter of the microneedles.

### 2.6. XPS

X-ray photoelectron spectroscopy is a surface-sensitive technique that was used to assess the chemical composition and the presence of impurities of the microneedle surfaces [24,25,26]. In this study, XPS was used to assess the chemical composition of the microneedle surfaces that were in contact with PDMS during the micromolding fabrication procedure.

### 2.7. Fourier Transform Infrared Spectroscopy

The Fourier transform infrared spectra were obtained using a Nexus 470 system; this system contains a continuum microscope, an OMNI sampler, and OMNIC^TM^ analysis software (Thermo Fisher, Waltham, MA, USA). The FTIR spectra of the microneedles were recorded from 400 to 4000 ${\mathrm{cm}}^{-1}$ with 1 ${\mathrm{cm}}^{-1}$ resolution [25].

### 2.8. Raman Spectroscopy

Raman spectroscopy is a common technique for chemical analysis [26,27]. Raman spectroscopy is used for determining the chemical properties of the microneedle tips and base sides (Renishaw, Gloucestershire, UK). A He-Ne laser with 633 nm wavelength was used to obtain the Raman spectra. The wavelengths used for the measurements were between 900 cm^−1^ and 1800 cm^−1^.

### 2.9. High Performance Liquid Chromatography

A Shimadzu HPLC (Kyoto, Japan) was used for determining the drug dosage in each MN; a Luna 5 µm 150 × 4.6 mm C18 column was used in this study. The mobile phase for the chromatographic separation of amphotericin B was N,N,N′,N′-tetramethyl ethylenediamine:acetonitrile (65:35 *v*/*v*); a 1.1 mL/min isocratic flow rate was used in this study. An injection volume of 50 μL was used in the study. The retention time for amphotericin B was 4.497 min; detection was performed using an ultraviolet light detector with 406 nm wavelength. Needles were broken by hand, and dissolved in methanol/DMSO (1:1 *w*/*w*), and sent for analysis by an external provider (Fungus Testing Laboratory, UT Health San Antonio, San Antonio, TX, USA). Five samples were prepared for HPLC testing; they were stored in a freezer before shipping.

### 2.10. Franz Diffusion Test

A Franz diffusion cell with a 12 mL receptor was used in this study. The pork skin was cut into the appropriate shape for the cell and equilibrated for 30 min in phosphate-buffered saline (PBS) (pH = 7.4). The receptor of Franz diffusion cell was filled with PBS (pH = 7.4); this value is aligned with human extracellular fluid pH value [28]. Pig skin was then placed in the cell. Next, 8% amphotericin B Gantrez^TM^ microneedles were made by hand to pierce the pig skin; they were left at room temperature for 6 h. All of the pig skin and the receptor PBS were homogenized with a homogenizer (IKA, T18, 115VAC) at 15,000 rpm for 30 min and filtered with the 0.45 µm pore membrane. The amphotericin B concentration is determined by HPLC; this process was repeated five times. This test was performed to understand the amount of drug delivered to the skin.

### 2.11. Antifungal Testing

An agar-based disk diffusion assay was used to assess the growth-inhibiting properties of amphotericin-B-loaded Gantrez^TM^ biodegradable copolymer microneedles toward the opportunistic fungal pathogen *Candida albicans* (ATCC 90028; American Type Culture Collection, Manassas, VA, USA) [27]. The reagents used for the fungal culturing process included yeast nitrogen base, dextrose, Sabouraud dextrose agar, and phosphate-buffered saline (10×) (VWR International, West Chester, PA, USA). Overnight broth cultures of *C. albicans* in yeast nitrogen base (YNB) and 100 mmol/L dextrose were made. Cell pellets were created via centrifugation (4500 rpm) for 10 min. The pellets were then resuspended to generate a cell density of approximately 10^8^ cells/mL in phosphate-buffered saline (PBS) (1×). PBS (10×) was diluted using deionized water to formulate PBS (1×). Sterile swabs were utilized to inoculate Sabouraud dextrose agar plates with *C. albicans* lawns. Microneedle samples were inverted and then placed directly onto the inoculated agar plate surface and incubated for 24 h at 37 °C. After 24 h, digital images of the agar plates were obtained and examined for regions of inhibited fungal growth (e.g., zone of inhibition (ZOI) measurements). For these modified agar tests, a 20–30 mm void area was removed from the Sabouraud dextrose agar; microneedles were seated or dispensed (for fully solvated samples) into the void area to prevent them from migrating across the agar surface during the 24 h incubation period.

Antifungal solution assessments were performed by solvating microneedles in 3.0 mL of PBS (1×) (pH = 7.4) for 24 h and subsequently preparing a 1:2 dilution series of the fully solvated solutions (100%) down to 1/64 of the original concentration. 1.0 mL of each dilution was then mixed with 1.0 mL of a 1:1000 suspension of *C. albicans* in YNB + 100 mmol/L dextrose to obtain a final effective microneedle concentration range of 100% to 0.78%. 0.15 mL of *C. albicans* inoculated dilutions were transferred in triplicate to a 96-well plate and allowed to incubate at a temperature of 37 °C at 150 rpm of shaking for 24 h. Absorbance measurements (at a wavelength of 600 nm) were subsequently collected to quantify solution growth and plotted as a function of microneedle solution concentration.

## 3. Results and Discussion

Microneedles may transform the delivery of many types of drugs [29,30]. Several criteria must be met for the delivery of a drug via microneedles, including appropriate mechanical properties. An appropriate matrix surface tension and viscosity are necessary for filling the molds completely. Higher viscosity and surface tension values lead to incomplete microneedles. Many solutions for preparing microneedles via molding have been described; for example, McGrath used a spray approach for producing microneedles and formed sharp microneedles with complete fidelity to the mold [31].

In this study, amphotericin-B-loaded Gantrez^TM^ AN-119 BF microneedles were fabricated using the micromolding technique. The master structure was fabricated by 2PP printing. The array shape is hexagonal, and needles are conical. Two different ratios amphotericin B–Gantrez^TM^ AN-119 BF ratios were assessed. In vitro studies demonstrated the antifungal properties of the fabricated microneedles.

**Figure 1:**
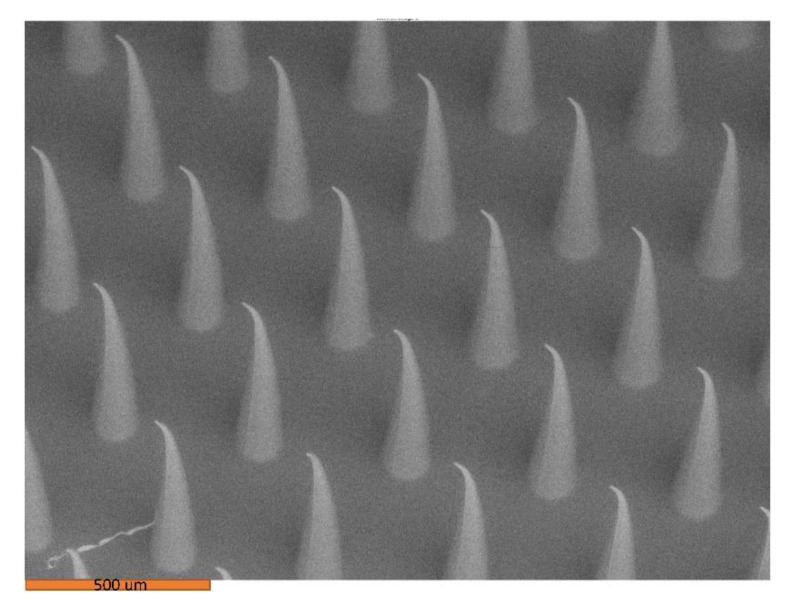
Scanning electron microscope image of amphotercin B–Gantrez^TM^ microneedles with buckled tips.

Figure 1 shows the buckled tips of Gantrez^TM^ AN-119 BF microneedles that resulted from heating for 5 h. When the PDMS molds are cured at 60 °C, the master structure tips are delicate and thin, which leads to tip buckling as shown in Figure 1.

**Figure 2:**
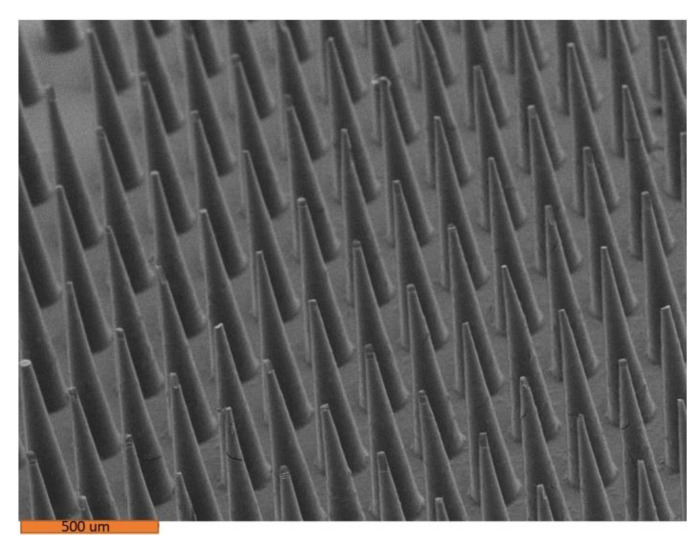
Scanning electron microscope image of 8% amphotericin B–Gantrez^TM^ microneedles.

**Figure 3:**
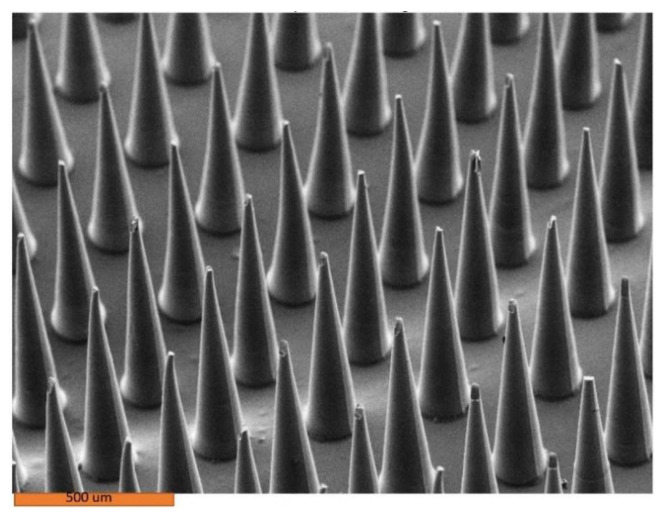
Scanning electron microscope image of 4% amphotericin B–Gantrez^TM^ microneedles.

Figure 2 and Figure 3 show the microneedles made in molds that were cured at room temperature. There is no buckling in these microneedles. It appears that heat, not human error, led to challenges with removal of microneedles from the mold.

Microneedles should be strong enough to pierce the first layer of skin. Park et al. suggested that the Young’s modulus of the microneedle material should exceed 1 GPa for skin penetration [32]. There are different types of variations that affect polymer mechanical properties, such as polymerization process temperature and the incorporation of various copolymers and cross-linkers [33,34]. The mechanical properties of needles and simulated needle failure under compression have been previously investigated [35]. For example, Du et al. determined the mechanical strengths of two types of the hyaluronic acid microneedles with and without drugs; they showed that that polymer molecular weight and amount of loaded drug affected microneedle mechanical behavior [36].

**Figure 4:**
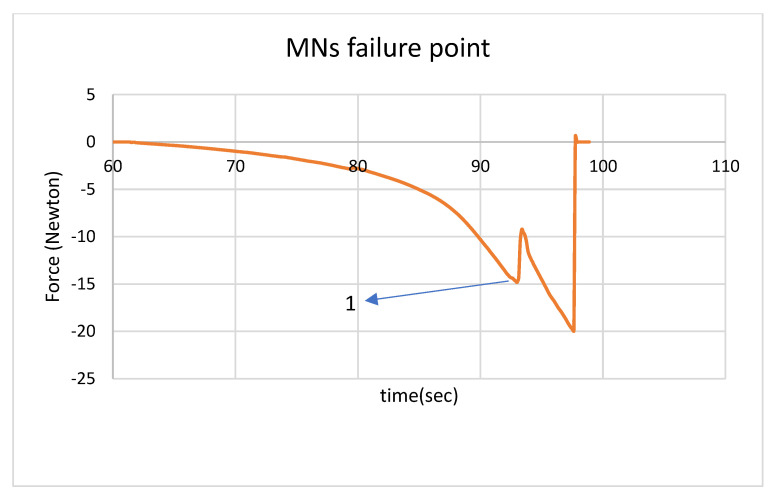
The load per time diagram of ElectroForce^®^ compression testing of a microneedle; point (1) reveals when the needle tips break.

Previous efforts were undertaken to understand the effect of geometry and supporting substrate attributes on microneedle penetration depth; for example, an ElectroForce^®^ 3100 instrument was previously used by Boonma et al. to assess the mechanical properties of sharp- and blunt-bevel microneedles [37]. For determining the fracture properties of the needles, an ElectroForce^®^ load–displacement instrument was used. When needles break under an increasing compressive load, the load value drops (as shown in Figure 4). Point (1) of Figure 4 shows that when the needle tips break, the height of the needles is decreased, and a sudden decrease in the load is observed. Table 1 shows compression of microneedles with two drug concentrations; the negative value of the load value indicates the compressive nature of the loading activity. On average, a needle with an 8% drug concentration failed at 0.54 N; a needle with a 4% drug concentration failed at 0.65 N. The addition of drug appears to reduce the compression properties of the polymer [31].

Nanoindentation was used to obtain hardness and Young’s modulus measurements [38]. Table 1 shows the mechanical properties of the microneedles with two different drug ratios. The Young’s modulus and failure force values were 8.65 GPa and 0.31 GPa, respectively, for the 4% drug concentration and 7.05 GPa and 0.43 GPa, respectively, for the 8% drug concentration. The Young’s modulus and failure force values for the 4% drug microneedles were slightly higher than for the 8% drug microneedles. The Young’s modulus and failure force values were decreased by increasing the amount of drug.

**Figure 5:**
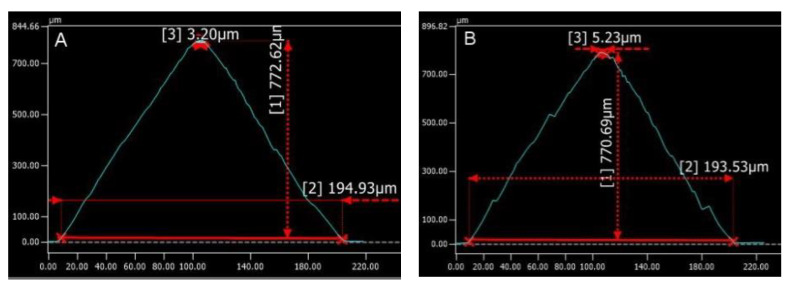
Keyence imaging results for (**A**) 4% amphotericin-B-loaded Gantrez^TM^ microneedles and (**B**) 8% amphotericin-B-loaded Gantrez^TM^ microneedles. The red lines represent measurements of the microneedle dimensions. Measurement [1] represents microneedle height, measurement [2] represents the microneedle base diameter, and measurement [3] represents microneedle tip diameter.

Figure 5 shows the needle tip and base diameters and the heights of two types of microneedles. The tip diameter, base diameter, and height for the 8% Amphotericin B Gantrez^TM^ AN-119 BF microneedles were 5.23, 193.53, and 770.69 μm, respectively. The tip diameter, base diameter, and height for 4% Amphotericin B Gantrez^TM^ AN-119 BF microneedles were 3.20, 194.93, and 772.62 μm, respectively. Changing the drug ratio will modify the viscosity, which may alter the microneedle’s sharpness and shape. The monomer matrix cannot easily penetrate the bores of the mold; increasing the drug ratio enhances the viscosity and makes penetration of the matrix into the mold bore more difficult [31]. The tips of the 4% drug microneedles are negligibly smaller, and the heights are negligbly taller. There was a greater amount of variation in the profile of 8% amphotericin-B-loaded microneedle; the roughness of the surface of this microneedle appears to be higher than the roughness of the surface of the 4% amphotericin-B-loaded microneedle.

**Figure 6:**
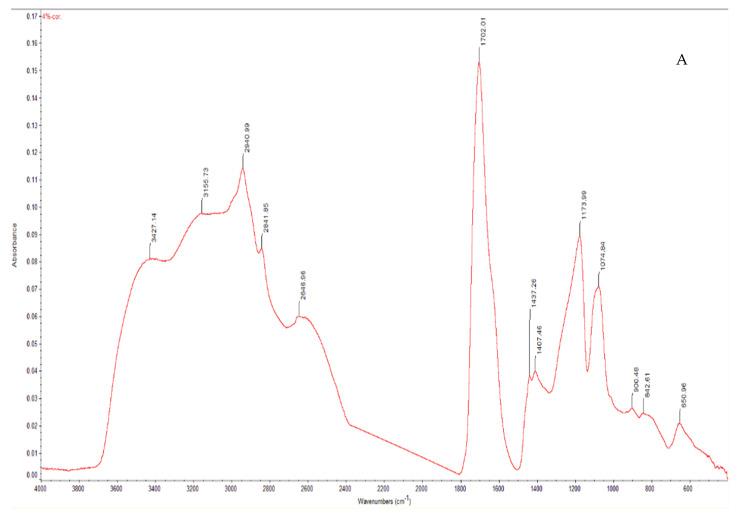

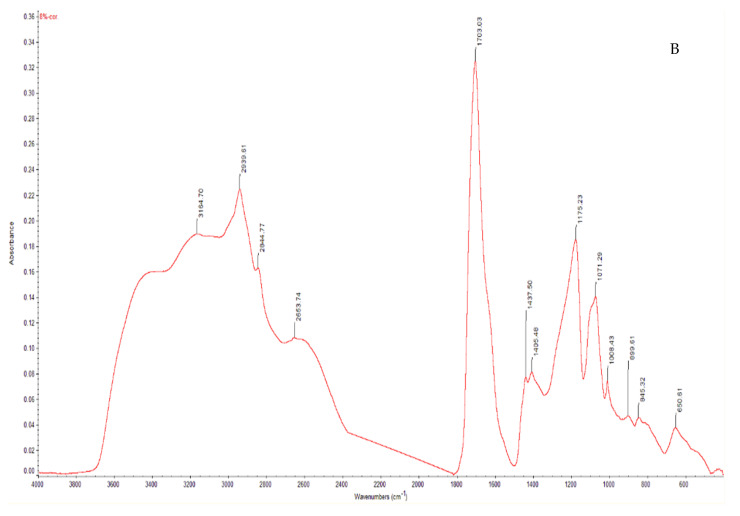
FTIR spectra from (**A**) 4% amphotericin B-loaded microneedles, and (**B**) 8% amphotericin B-loaded microneedles.

FTIR is often used to understand the structure of polymeric microneedles [39]. Figure 6 demonstrates the FTIR results of the needles made with amphotericin B and Gantrez^TM^ AN-119 BF. The contributions of amphotericin B to the spectra are associated with the following features: C=C stretching (at approximately 1625 cm^−1^), C–H stretching (at approximately 3025 cm^−1^), C–H (at approximately 840 cm^−1^), C–O stretching (at approximately 1407 cm^−1^), N–H (overlapped peak at approximately 650 cm^−1^), and O–H stretching (at approximately 3420 cm^−1^). Sachan et al. characterized amphotericin B microneedles with FTIR and observed similar spectral features [40].

**Figure 7:**
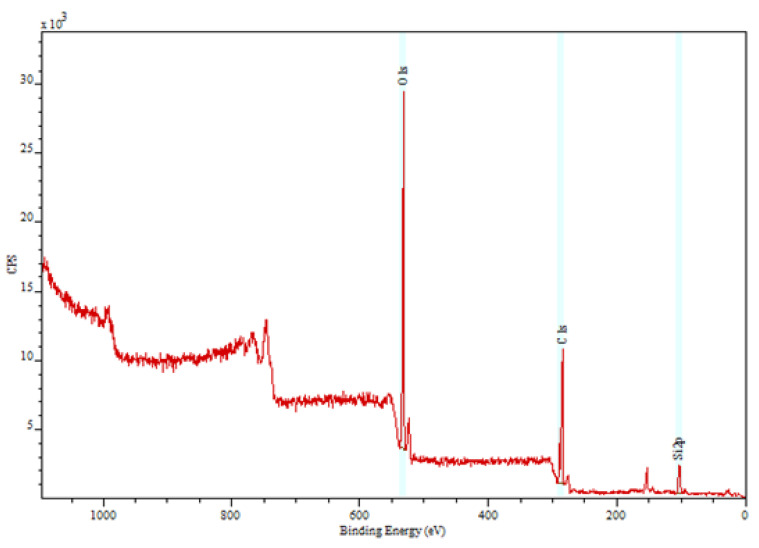
XPS spectrum of the combination of Gantrez^TM^ AN-119 BF and amphotericin B.

As shown by Boehm et al., X-ray photoelectron spectroscopy is useful for evaluating the chemical composition of new types of microneedles [41]. Figure 7 shows the XPS spectrum from amphotericin B-loaded Gantrez^TM^ AN-119 BF microneedles, which shows the presence of C (51.9%), O (38.0%), and Si (10.1%) on the surface. It is important to note that no Si atoms occur in either amphotericin B or Gantrez^TM^ AN-119 BF. The presence of Si atoms in the XPS data may be attributed to the contact between the microneedles and the PDMS mold. Fortunately, Si atoms should not impart toxicity to the microneedles [42]. Insertion of the MNs reduces skin barrier function and increases transepidermal water loss. The water loss will continue for several hours before the pores close and the skin barrier function is restored [19,43,44,45].

HPLC results showed the drug amount in each microneedle type. Three microneedles were tested; the results showed that each microneedle (8% concentration) was loaded with 0.85 mg of amphotericin B (standard deviation = 0.027). A Franz diffusion test showed that 88.88% of the loaded amphotericin B was delivered to skin (standard deviation = 0.0164). Since the patches are not flexible, the entire height of a needle did not permeate the skin and was not dissolved in the skin; however, the permeated component diffused into the Franz cell receptor fluid. Based on the literature, the extracellular fluid pH (approximately 7.4) is aligned with Franz cell receptor pH [28]. Gantrez^TM^ was noted to be soluble in PBS with a pH of 7.4, which suggests that Gantrez^TM^ will be soluble in extracellular fluid (e.g., interstitial fluid) with the same pH value [17].

**Figure 8:**
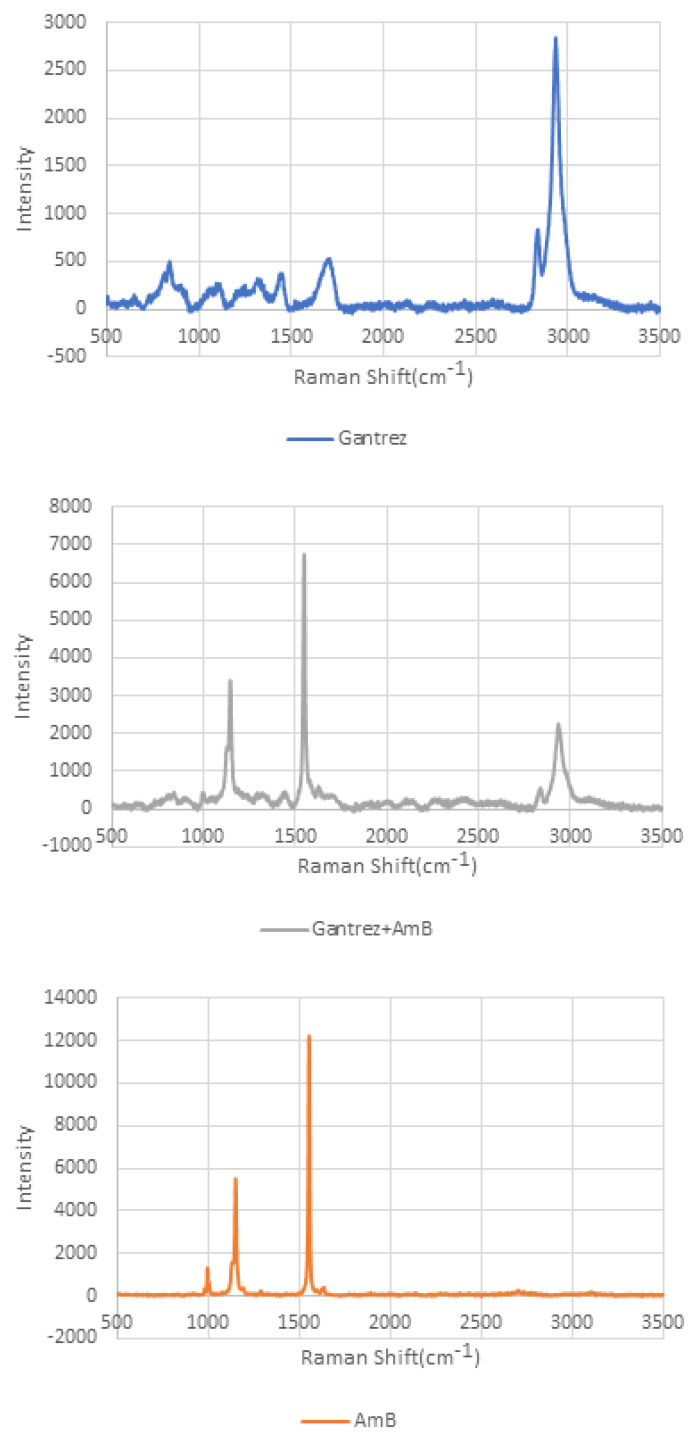
Raman spectrum of Gantrez^TM^/amphotericin B (**top**), amphotericin B (**center**), and Gantrez^TM^ (**bottom**).

A Raman spectroscopy microscope was used to study needles that were broken and placed on the microscope stage. Figure 8 shows the Raman intensity spectra for amphotericin B, Gantrez^TM^, and the combination of Gantrez^TM^ and amphotericin B [46,47]. The bands at 1445 cm^−1^ and 1702 cm^−1^ were assigned to CH_2_ and CH_3_ deformations, respectively. The band at 1836 cm^−1^ was assigned to hydrocarbon bonding (e.g., CH_3_); the bands at 2940 cm^−1^ and 2842 cm^−1^ were assigned to CH bonding.

**Figure 9:**
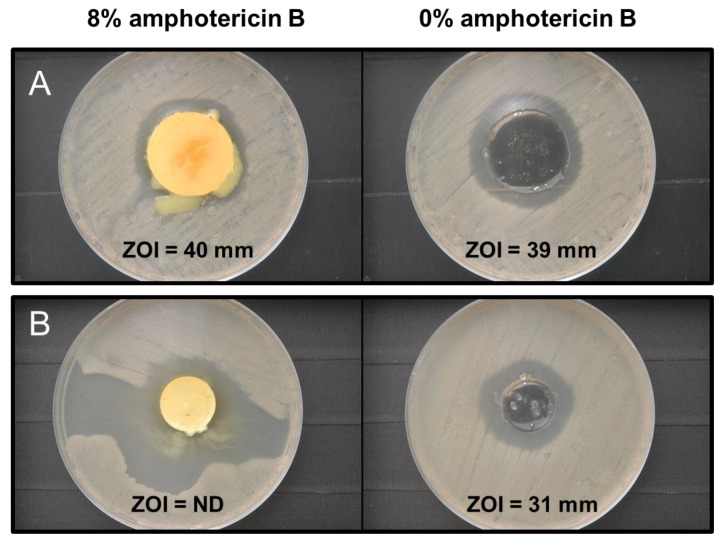
Modified disk diffusion assay results involving *Candida albicans* for 8% and 0% amphotericin-B-loaded Gantrez^TM^ microneedles; (**A**) partially solvated microneedles; (**B**) fully solvated microneedles. ZOI = zone of inhibition.

Antifungal testing of microneedles through the use of agar diffusion testing is shown in Figure 9. Initially, the microneedles were characterized by inverting and placing the samples directly in contact with agar inoculated with lawns of *C. albicans*, which resulted in migration of the samples across the agar surface toward the periphery of the petri dish during the 24 h incubation period at 37 °C as a consequence of hydration and partial solvation of the microneedles. To ameliorate this issue, the microneedles were seated into a 30 mm void area that was excised from the center of the agar slab to immobilize and prevent sample movement during the incubation period (Figure 9A). The measured zone of inhibition (ZOI) due to diffusion of antifungal components was similar for both the 8% and 0% amphotericin-B-loaded microneedles—40 mm and 39 mm, respectively. These results revealed that the Gantrez^TM^ biodegradable acid anhydride copolymer imparts a baseline level of antifungal activity to the microneedles. Interestingly, the Gantrez^TM^ microneedles loaded with 8% amphotericin B did not exhibit any substantive enhanced activity toward *C. albicans*.

To investigate the possibility that amphotericin B diffusion was impeded due to incomplete or only partial solvation of the samples, both 8% amphotericin-B-loaded microneedles and 0% amphotericin-B-loaded microneedles were allowed to fully solvate in 3 mL of PBS (1×) at 37 °C for 20 h prior to dispensing 0.7 mL of the fully solvated samples into agar void areas (20 mm). Using this modified agar diffusion testing protocol, the 8% amphotericin-B-loaded microneedles demonstrated enhanced antifungal activity relative to the pure Gantrez^TM^ biodegradable copolymer (0% amphotericin B) as evidenced qualitatively by its larger area of *C. albicans* growth inhibition (Figure 9B). The irregular nature of this region, however, precluded a subsequent ZOI measurement.

**Figure 10:**
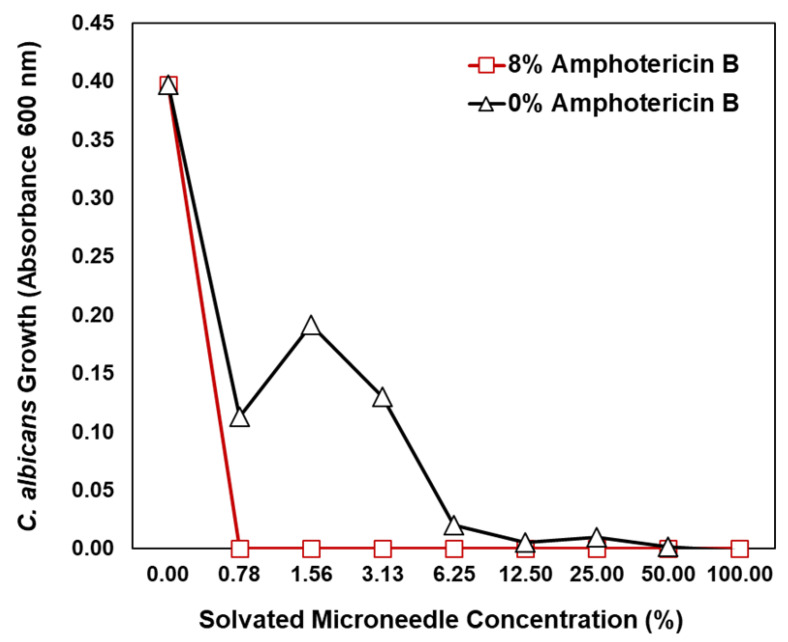
*Candida albicans* solution growth assessments in nutrient media dilutions of fully solvated microneedles. The 100% concentration denotes microneedle samples solvated in 3 mL of PBS (1×).

In addition to agar growth assessments, microneedles fully solvated in PBS (1×) were characterized by their ability to prevent planktonic growth of *C. albicans* in solution (Figure 10). A 1:2 dilution series of the fully solvated microneedle samples in nutrient growth media was prepared and evaluated, yielding an effective concentration range of 100% (i.e., fully solvated polymer) down to 0.78%. Complete growth inhibition of *C. albicans* was observed at each concentration of the solvated 8% amphotericin-B-loaded microneedles tested. In contrast, *C. albicans* growth was reduced by only 50–70% when cultured in the three lowest 0% amphotericin-B-loaded microneedle concentrations (0.78%, 1.56%, and 3.13%) and was completely inhibited at only the two highest concentrations assessed (50% and 100%). The solution testing data, taken in conjunction with the modified agar diffusion results, clearly demonstrate the enhanced antifungal properties of 8% amphotericin-B-loaded microneedles relative to the non-loaded, pure Gantrez^TM^ biodegradable copolymer analog.

## 4. Conclusions

In this study, amphotericin-B-loaded Gantrez^TM^ AN-119 BF microneedles were fabricated using a micromolding technique; amphotericin B, an antifungal drug, was incorporated in the devices. The master structure for mold fabrication was created by two-photon polymerization. Molds were fabricated by PDMS under two different thermal situations. The tips in the master structure were sharp; higher temperatures caused these features to buckle. PDMS cured at room temperature provided sharper Gantrez^TM^ AN-119 BF microneedles. Nanoindentation and compression testing results showed that the microneedles possessed appropriate mechanical properties for penetrating the topmost layer of the skin. Nanoindentation was used to obtain hardness and Young’s modulus values for the mixed Gantrez^TM^ AN-119 BF/amphotericin B polymer; compression testing demonstrated that the failure force of the needles under compressive loading was sufficient for penetration of the topmost layer of the skin. Confocal microscopy confirmed the sharpness of the needles. The chemical properties of the needles were demonstrated using Raman spectroscopy, FTIR, and XPS. XPS showed the presence of Si on the needles’ surface, which may have been introduced by the PDMS molds. Finally, in vitro agar diffusion studies demonstrated the antifungal properties of the microneedles. The advantages of these microneedles include the loading of a relatively high volume, 0.85 mg of AmB, in the MNs. One disadvantage of the AmB MNs includes the potential effect of the MN material on drug stability, including drug stability over extended periods of time; additional efforts are anticipated to understand the effect time and temperature on the stability of the MNs.

## Figures and Tables

**Table 1:** Mechanical properties of amphotericin-B-loaded Gantrez^TM^ polymer from nanoindentation and ElectroForce^®^ mechanical testing.

Drug Concentration	Er (GPa)	Standard Deviation	H (GPa)	Standard Deviation	Newton/Needle	Standard Deviation
4%	8.65	1.1	0.31	0.85	0.65	2.02
8%	7.05	1.23	0.43	0.96	0.54	2.25

## Data Availability

The datasets generated during and/or analyzed during the current study are available from the corresponding author on reasonable request.
